# RETRACTION: Ultrasound in Paediatric Surgery: A Meta‐Analysis Review of its Influence on Postoperative Wound Healing and Infection Rates

**DOI:** 10.1111/iwj.70602

**Published:** 2025-04-21

## Abstract

**RETRACTION:** PengY. , LiuQ. , TangJ. , ZhouM. , LiuLP. , and LiuJ. , “Ultrasound in Paediatric Surgery: A Meta‐Analysis Review of its Influence on Postoperative Wound Healing and Infection Rates,” International Wound Journal 21, no. 3 (2024): e14462, 10.1111/iwj.14462.37931597 PMC10898372

The above article, published online on 06 November 2023, in Wiley Online Library (http://onlinelibrary.wiley.com/), has been retracted by agreement between the journal Editor in Chief, Professor Keith Harding; and John Wiley & Sons Ltd. Following an investigation by the publisher, all parties have concluded that this article was accepted solely on the basis of a compromised peer review process. The editors have therefore decided to retract the article. The authors did not respond to our notice regarding the retraction.

**RETRACTION:** Y. Peng , Q. Liu , J. Tang , M. Zhou , LP. Liu , and J. Liu , “Ultrasound in Paediatric Surgery: A Meta‐Analysis Review of its Influence on Postoperative Wound Healing and Infection Rates,” International Wound Journal 21, no. 3 (2024): e14462, 10.1111/iwj.14462.37931597 PMC10898372

The above article, published online on 06 November 2023, in Wiley Online Library (http://onlinelibrary.wiley.com/), has been retracted by agreement between the journal Editor in Chief, Professor Keith Harding; and John Wiley & Sons Ltd. Following an investigation by the publisher, all parties have concluded that this article was accepted solely on the basis of a compromised peer review process. The editors have therefore decided to retract the article. The authors did not respond to our notice regarding the retraction.

